# Operationalizing resilience for adaptive coral reef management under global environmental change

**DOI:** 10.1111/gcb.12700

**Published:** 2014-09-05

**Authors:** Kenneth RN Anthony, Paul A Marshall, Ameer Abdulla, Roger Beeden, Chris Bergh, Ryan Black, C Mark Eakin, Edward T Game, Margaret Gooch, Nicholas AJ Graham, Alison Green, Scott F Heron, Ruben van Hooidonk, Cheryl Knowland, Sangeeta Mangubhai, Nadine Marshall, Jeffrey A Maynard, Peter McGinnity, Elizabeth McLeod, Peter J Mumby, Magnus Nyström, David Obura, Jamie Oliver, Hugh P Possingham, Robert L Pressey, Gwilym P Rowlands, Jerker Tamelander, David Wachenfeld, Stephanie Wear

**Affiliations:** 1 Australian Institute of Marine SciencePMB3, Townsville, Qld, 4810, Australia; 2 Great Barrier Reef Marine Park AuthorityPO Box 1379, Townsville, Qld, 4810, Australia; 3 International Union for the Conservation of Nature, Gland Switzerland and University of QueenslandBrisbane, Qld, Australia; 4 The Nature ConservancyBig Pine Key, FL, USA; 5 Department of the Environment, Great Barrier Reef TaskforceAllara St, Canberra, ACT, Australia; 6 Coral Reef Watch, US National Oceanic and Atmospheric AdministrationCollege Park, MD, USA; 7 The Nature ConservancyWest End, Qld, 4101, Australia; 8 Australian Research Council Centre of Excellence for Coral Reef Studies, James Cook UniversityTownsville, Qld, Australia; 9 Coral Reef Watch, National Oceanographic and Atmospheric Administration and School of Engineering and Physical Sciences, James Cook UniversityTownsville, Qld, Australia; 10 Atlantic Oceanographic and Meteorological Laboratory, National Oceanographic and Atmospheric AdministrationMiami, FL, USA; 11 Spatial Ecology Lab, University of QueenslandSt Lucia, Qld, 4072, Australia; 12 Fiji Country Program, Wildlife Conservation Society11 Ma'afu Street, Suva, Fiji; 13 CSIRO Ecosystem SciencesPMB, Aitkenvale, Qld, 4810, Australia; 14 USR 3278 CNRS-EPHE, CRIOBEBP 1013 Papetoai, Moorea, 98729, Polynesie Francaise; 15 The Nature ConservancyAustin, TX, USA; 16 Stockholm Resilience Centre, Stockholm UniversityKräftriket 2B, Stockholm, SE-104 05, Sweden; 17 CORDIO East AfricaP.O.Box 10135-80101, Mombasa, Kenya; 18 Australian Research Council Centre of Excellence for Environmental Decisions, University of QueenslandSt Lucia, Qld, Australia; 19 National Coral Reef Institute, Nova Southeastern UniversityDania Beach, FL, USA; 20 UN, United Nations Environment ProgrammeRajdamnern Nok Av., Bangkok, 10200, Thailand; 21 The Nature Conservancy, Department of Biology, University of FloridaGainesville, FL, USA

**Keywords:** climate change, coral reefs, ecosystem vulnerability, environmental management, ocean acidification, social-ecological system, structured decision-making

## Abstract

Cumulative pressures from global climate and ocean change combined with multiple regional and local-scale stressors pose fundamental challenges to coral reef managers worldwide. Understanding how cumulative stressors affect coral reef vulnerability is critical for successful reef conservation now and in the future. In this review, we present the case that strategically managing for increased ecological resilience (capacity for stress resistance and recovery) can reduce coral reef vulnerability (risk of net decline) up to a point. Specifically, we propose an operational framework for identifying effective management levers to enhance resilience and support management decisions that reduce reef vulnerability. Building on a system understanding of biological and ecological processes that drive resilience of coral reefs in different environmental and socio-economic settings, we present an Adaptive Resilience-Based management (ARBM) framework and suggest a set of guidelines for how and where resilience can be enhanced via management interventions. We argue that press-type stressors (pollution, sedimentation, overfishing, ocean warming and acidification) are key threats to coral reef resilience by affecting processes underpinning resistance and recovery, while pulse-type (acute) stressors (e.g. storms, bleaching events, crown-of-thorns starfish outbreaks) increase the demand for resilience. We apply the framework to a set of example problems for Caribbean and Indo-Pacific reefs. A combined strategy of active risk reduction and resilience support is needed, informed by key management objectives, knowledge of reef ecosystem processes and consideration of environmental and social drivers. As climate change and ocean acidification erode the resilience and increase the vulnerability of coral reefs globally, successful adaptive management of coral reefs will become increasingly difficult. Given limited resources, on-the-ground solutions are likely to focus increasingly on actions that support resilience at finer spatial scales, and that are tightly linked to ecosystem goods and services.

## Introduction

### The need for adaptive resilience-based management of coral reefs

Natural resource managers are facing growing challenges from multiple and cumulative stressors that are increasing the vulnerability of ecosystems and societies that depend on their goods and services (Chapin *et al*., 2000). Coral reefs are vulnerable to the global pressures of climate change and ocean acidification (Hoegh-Guldberg *et al*., 2007; Moss *et al*., 2010) and to a suite of regional and local-scale disturbances including destructive fishing and overfishing, poor coastal and urban development and pollution (Knowlton & Jackson, 2008).

The management challenges associated with coral reef vulnerability include at least two key facets: (i) reducing pressures and exposures to stress, and (ii) support of the system's resilience to these threats. Coral reef managers are increasingly shifting their focus from strictly stress abatement to including a broader support of ecosystem resilience – i.e. supporting ecosystem processes that lower sensitivity, promote recovery and enhance adaptive capacity (e.g. Marshall & Shuttenberg, 2006; GBRMPA, 2009; McClanahan *et al*., 2012). This shift has been reinforced by an increase in adaptive management efforts and the implementation of systems approaches to management and conservation (e.g. Chapin *et al*., 2010; McCook *et al*., 2010). Resilience provides an important framework for these more integrated and dynamic approaches and helps managers deal with the combined and often synergistic impacts of global and local stressors (Tompkins & Adger, 2004).

Climate change and ocean acidification scenarios for this century (Cao *et al*., 2007; Moss *et al*., 2010) are expected to challenge the natural resilience of tropical coral reefs (Anthony *et al*., 2011). This is in part driven by increased coral bleaching risk (van Hooidonk & Huber, 2009; van Hooidonk *et al*., 2013), increased storm intensity (Knutson *et al*., 2010; Emanuel, 2013), increased reef fragility to storms (Madin *et al*., 2008) and reduced coral growth (Reynaud *et al*., 2003) and recovery rates (Hoegh-Guldberg *et al*., 2007; Albright & Langdon, 2011). From a reef management and policy perspective, this means that climate change and ocean acidification will, firstly, increase the need for efforts to abate regional- and local-scale stressors (i.e. those open to on-the-ground management intervention) on coral reefs, increase the vulnerability of reef-dependent people and, thirdly, increase the need to enhance ecosystem resilience (Kennedy *et al*., 2013).

Adaptive resilience-based management (ARBM) was developed from studies of the dynamics of linked social and ecological systems (Anderies *et al*., 2006) and has influenced systems thinking of managers across terrestrial, freshwater and marine systems (Chapin *et al*., 2010; Rist *et al*., 2013). Despite ARBM being a recommended approach for coral reefs (Hughes *et al*., 2010; Graham *et al*., 2013) and readily incorporated into management documents (e.g. GBRMPA, 2009), there are few examples of practical implementation of resilience principles in the adaptive management and decision-making on coral reefs (Maynard *et al*., 2010; Weeks & Jupiter, 2013).

The key objective of this paper is to present a framework that can help reef managers and conservation practitioners identify viable intervention options and make effective decisions to reduce coral reef vulnerability under complex environmental and social scenarios based on a complex systems understanding. We argue that practical implementation of ARBM could be enhanced through an approach that more formally integrates key principles of ecosystem vulnerability, ecological resilience, disturbance regimes, management options and structured decision-making. We then present mechanisms by which resilience principles can be made operational (sensu Sarkar & Margules, 2002) to support the adaptive management of coral reefs and dependent societies under regional and global environmental change.

### The concepts of resilience and vulnerability in the context of managing social-ecological systems

Supporting ecosystem resilience provides opportunities to enhance the system's ability to cope with extrinsic pressures (including those beyond the direct influence of coral reef managers), and to reorganize and/or recover between disturbances, thereby reducing the vulnerability of the ecosystem and dependent societies. We use the ecological resilience definition to describe ecosystem resilience, broadly defined as the capacity of a system to absorb disturbances and reorganize, while undergoing change so as to still retain essentially the same function, structure, identity, and feedbacks (Holling, 1973; Gunderson, 2000; Nyström *et al*., 2008). Within coral reef ecosystems, ecological resilience is the result of biological and ecological processes facilitating recruitment, regrowth, repair and reassembly. These processes occur along multiple dimensions including levels of organization, trophic structure (Bellwood *et al*., 2004), time (Anthony *et al*., 2011) and space (Nyström & Folke, 2001). Resistance, which is the capac-ity to withstand disturbances such as storm damage, and recovery from such disturbances, are both components of ecological resilience (Nyström *et al*., 2008).

Resilience has also been a formative concept in understanding dynamics and trajectories of social systems. Similar to ecological resilience, social resilience describes the capacity of societies and individuals to cope and adapt to change, and it often depends on the existence of institutions that learn and store knowledge, and which are creative and flexible in approaching problems (Gunderson & Holling, 2002). Importantly, and based on resilience theory, social and ecological systems are often intrinsically coupled and constantly face change together. Consequently, managing resilience of the linked ‘socio-ecological’ system is a way to integrate and manage the interactions and feedbacks between people and nature (Berkes & Folke, 1998; Chapin *et al*., 2010; Folke *et al*., 2010).

Ecosystem vulnerability is the risk that average state of the system falls to an unacceptable level (Mumby *et al*., 2014). Broadly, vulnerability is defined as the product of three key system properties: (i) exposure to stressors, pressures or disturbances, (ii) sensitivity (or lack of resistance) to such exposure and (iii) the capacity to adapt to and/or recover from disturbances (Füssel & Klein, 2006; Marshall *et al*., 2013). If low ecosystem vulnerability is the fundamental management objective, then it can be achieved via actions to (i) reduce exposure, (ii) support resilience (resistance and recovery/adaptive capacity) of the linked socio-ecological system, or (iii) both.

As climate change and ocean acidification unfold, increasing the exposure of marine ecosystems to a suite of global stressors, the vulnerability of coral reefs is expected to increase via eroded resilience (Anthony *et al*., 2011; Mumby *et al*., 2014) and enhanced disturbance regimes (Hoegh-Guldberg *et al*., 2007). Thus, viable management options and effective actions to reduce reef vulnerability to a variety of stressors will require considerations of a growing set of innovative management alternatives that can both tackle stressors and enhance ecosystem resilience locally or regionally (Game *et al*., 2014). Importantly, however, there are limits to an ecosystem's natural resilience (Thrush *et al*., 2009), and managers need to take those limits into account. In the following we introduce and review resilience models and drivers of resilience processes to first provide a system's context for adaptive coral reef management under environmental change.

### Resilience models – stability landscapes

**Fig 1:**
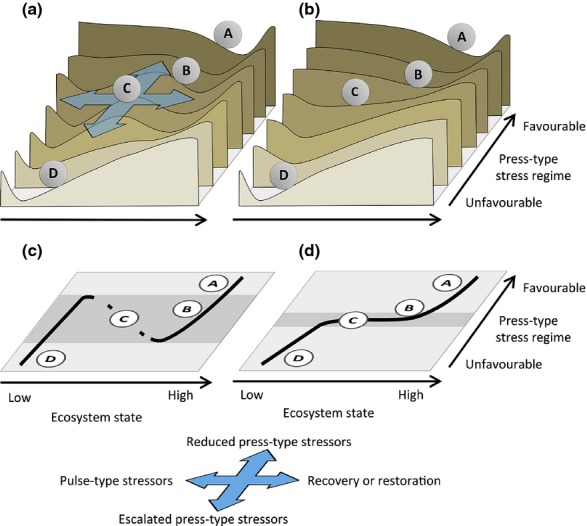
Examples of ecosystem stability landscapes, based on the conceptual model by Scheffer *et al*. (2001), illustrating the change in system dynamics as a function of system state and environmental conditions (press-type stress regime). Panel (a) represents coral reef ecosystems that show bistable states (e.g. Caribbean reefs), and panel (b) represents reefs that show single equilibrial states (e.g. Indo-Pacific reefs, Roff & Mumby, 2012). The dynamics of the system (represented by the behaviour of a ball) are determined by four sets of forces: (i) pulse-type stressors, (ii) recovery processes and active restoration, (iii) press-type stressors leading to declining environmental conditions and (iv) improvement in press-type conditions. Resilience is largely characterized by the shape of the landscape slice under a given environmental condition (press-type stress regime). Note that scales on *y*-axes are not comparable between models.

Stability landscapes (Scheffer *et al*., 2001; Scheffer & Carpenter, 2003) provide a useful conceptual representation of ecosystem resilience for coral reefs (Hughes *et al*., 2010) and how different stressors affect ecosystem behaviour. In essence, stability landscapes are three-dimensional representations of how a system (indicated by a ball) gravitates towards system equilibria (bottom of valleys) following disturbances (pulse-type stressors, see below) within a space described by ecosystem state (*x*-axis) and environmental conditions (press-type stress regimes, *y*-axis, Fig.1). In this representation, resilience is proportional to valley depth in the state dimension (Scheffer *et al*., 2012) and the height of hills forming barriers to the system transitioning into another gravitational basin (e.g. from corals to macroalgae; Bellwood *et al*., 2004) following a pulse-type disturbance. Simulations and analytical models of coral reef dynamics based on empirical data demonstrate that the characteristics of stability landscapes are shaped by ecosystem processes, and by their interactions and feedbacks between stressors and processes (Mumby *et al*., 2007; Anthony *et al*., 2011; Scheffer *et al.,* 2012). Importantly, because ecosystem dynamics and processes are associated with substantial uncertainty, the location of gravitational basins, equilibria and thresholds on stability landscapes must be viewed as probabilistic and used to provide guidelines only.

Reefs with different tendencies to form alternate stable states (Mumby *et al*., 2012; Roff & Mumby, 2012) display different stability landscapes (Scheffer *et al*., 2001). Coral reef systems displaying alternate stable states between coral and macroalgae have only been demonstrated unequivocally for Caribbean systems; Indo-Pacific systems tend to display single equilibrial states (Roff & Mumby, 2012; but see also Cheal *et al*., 2013). For representation, we base our examples of ARBM on two contrasting types of stability landscapes: one with a pronounced tendency to form alternate stable states (e.g. corals and macroalgae forming opposite but simultaneous basins of attraction), and one with only a single equilibrial state for a given set of environmental conditions (e.g. *either* corals *or* macroalgae forming a gravitational basin, Fig.1). Thresholds (conditions representing increased probability of abrupt shifts between contrasting states) exist in both types (Scheffer & Carpenter, 2003), but have different risk implications (Fig.1). First, systems displaying alternate stable states have two environmental thresholds. One marks the transition between a coral-dominated regime and a coral-macroalgae bistable regime. This is indicated by the transition from condition A to B in Fig.1a, crossing the upper edge of the shaded zone in Fig.1c. The other marks the transition from the bistable regime into an algal-dominated one. This is shown as a transition from scenario C to D in Fig.1a, and a move out of the shaded zone in Fig.1c. This example is typical of Caribbean reef systems where the locations of dynamic thresholds along the press-type stress regime axis are functions of a suite of environmental pressures, most prominently water quality, algal growth rate and overfishing of herbivores (Mumby *et al*., 2007; Roff & Mumby, 2012).

Expanded models demonstrate that the locations of these thresholds are strongly affected by ocean warming and acidification (Anthony *et al*., 2011). Secondly, systems without a propensity to form alternate stable states display one, though dynamic, threshold, which marks an increased probability for shifts between coral- and macroalgal-dominated regimes. The crossing of this threshold is indicated in Fig.1b and d as a transition from scenario B to C across the narrow shaded zone. Again, the location of this threshold is strongly driven by the combination of press-type stressors, including ocean warming and acidification (Anthony *et al*., 2011). In a later section we show that the two types of stability landscapes have different implications for resilience-based management across environmental scenarios and geographical settings, but that a set of general rules apply to both.

**Fig 2:**
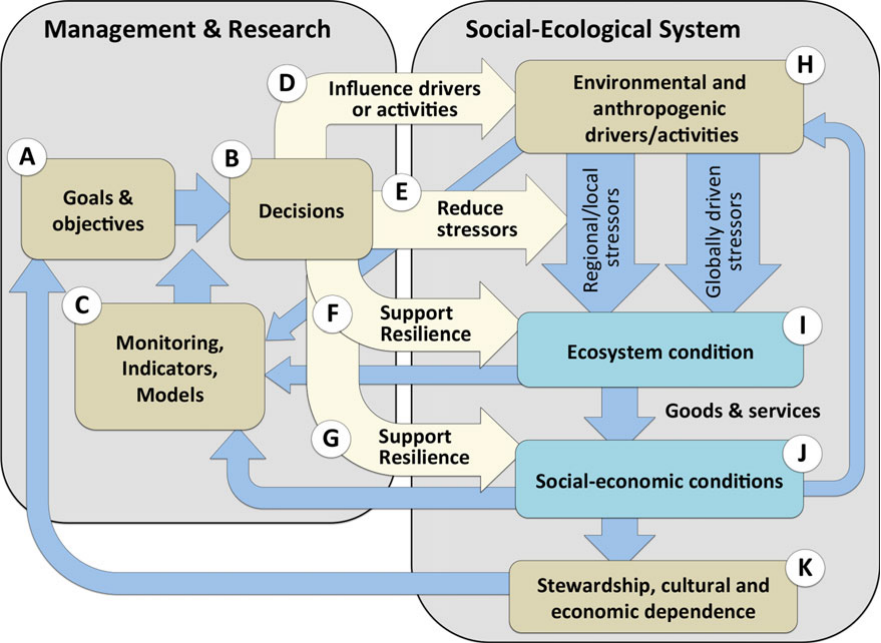
System diagram outlining the functional linkages within the operational adaptive resilience-based management (ARBM) framework, consisting of a management and research component (left box, A–G) and the stressors/activities/drivers and conditions of the social-ecological systems (right box, H–K). The system is dynamic as information flowing from the ecological and social systems is used to update objectives and specific decisions to intervene and manage drivers, activities or stressors influencing resilience processes.

### Environmental and human drivers of resilience

Understanding which environmental or anthropogenic stressors impact resilience, and which do not, is a critical basis for ARBM (Table1). A key functional categorization for understanding the implications of different stressors is that of pulse (acute) vs. press (chronic) (Scheffer *et al*., 2001). In the general working model for resilience in Fig.1, pulse-type stressors move the system state (the ball) from right to left (*x*-axis) over a short timeframe (acute disturbance events), while press-type stressors move the system downwards to less favourable conditions over longer timeframes. Depending on the system (i.e. the stability landscape and the location of thresholds), increased press-type stress can reduce resilience to pulse-type disturbances (e.g. a shift from scenario A to B or C in Fig.1a and b), in part by making the system more susceptible to pulse-type stressors. In the context of ARBM, processes of reef recovery or active restoration are forces directly opposing pulse-type stressors (Fig.1). Here, fast coral recovery, and potentially widespread restoration, can increase resilience by promoting gravitation towards equilibrium before the next pulse-type disturbance occurs. Conversely, reduced press-type stressors, for example through environmental management of water quality or overfishing, can enhance resilience by moving the system towards the safe side of the environmental threshold and into the coral-dominated regime where pulse-type disturbances are less likely to trigger a phase shift (e.g. from scenarios B to A).

**Table 1:** Key stressors on coral reefs, their pulse- vs. press-type characteristics and their role in adaptive resilience-based management (ARBM)

Stressor	Pulse/Press	Drivers or activities	Impact	Resilience processes affected	Potential management levers (see also Table2)
Storms	Pulse (stochastic)	Natural cycles, climate change	Structural damage, floods and sediment-ation	Recovery and connectivity if damage is extensive	Preparedness and recovery planning locally; compensatory measures
Destruct-ive fishing	Pulse	e.g. bomb fishing, poison fishing	Structural damage, mortality of flora and fauna	Recovery, reproduction, recruitment and connectivity if damage is extensive	Increase incentives for nondestructive harvest of resource through education, regulation and enforcement
Crown-of-thorns starfish (CoTS)	Pulse	Nutrient enrichment, natural cycles	Coral mortality	Recovery, recruitment and connectivity if mortality is extensive	Improved management of catchment, protection of CoTS predators, tactical CoTS control
Thermal anomalies	Pulse, with press-type after-effects	Climate change, natural cycles	Coral bleaching, diseases and mortality	Reduced growth and reproduction, and potentially connectivity if impact is extensive	Identify sites that may have lower vulnerability; protect from local stressors; manage for enhanced recovery
Sedimenta-tion/turbidity	Mixed depending on source	Mixed: land use and river catchment practices, flooding, resuspension, coastal construction	Sediment stress and light limitation, enhancement of algal growth	High turbidity from re-suspension can cause long-term suppression of coral recovery and provide competitive advantage to other benthic groups such as algae and sponges	Improved management of catchment land use through education, regulation, incentives and penalties. Restore land vegetation. Control coastal development activities.
Nutrient enrichment	Press, but pulse if linked to flood events	Mixed: land use and river catchment practices, flooding	Enhanced algal growth, increased turbidity	Increases susceptibility of corals to thermal bleaching. Provides competitive advantage to algae, which can suppress coral recovery.	Improved management of sewage and intensive agriculture activities through education, regulation, incentives and penalties
Pollution (herbicides, pesticides and heavy metals)	Press, but pulse if linked to flood events or marine incidents	Land-based (urban and agriculture) and from shipping	Toxicity, affects metamorphosis and larval survival.	Reduced coral growth and reproduction. Suppresses reef supply-side ecology.	Improved management of urban, agricultural and shipping activities through education, regulation, incentives and penalties
Ocean acidification	Press	Direct CO_2_ effect, point and nonpoint sources of low pH runoff	Reduced coral growth and strength, enhanced algal growth	Coral growth rates, skeletal strength and recruitment reduced.	Identify sites that could have lower vulnerability and target for protection from local stressors, control land-based sources of pollutants that decrease pH (e.g. nitrogen/sulfur oxides)
Decline in herbivores	Press	Human use	Reduced algal mortality, algal overgrowth of corals	Potentially drive phase shift to macroalgae, exacerbated by nutrients, warming and acidification	Improved fisheries management through education, regulation, incentives and penalties.

#### Pulse-type stressors

On coral reefs, pulse-type stressors include tropical cyclones, coral bleaching events, destructive fishing, crown-of-thorns starfish (CoTS) outbreaks, and flood events (Table1). These events may not impact directly on resilience processes, but they episodically send the system back to an earlier successional state (leftward system shift in Fig.1). In systems with high resilience under favourable conditions (scenario A in Fig.1), pulse events may not cause sustained reductions in ecosystem values if the system has time to recover or reorganize (rightward shifts) between events (Halford *et al*., 2004; Roff & Mumby, 2012). Pulse-type stress events that occur with high frequency or severity, however, increase the demand for fast recovery and reorganization, and hence the demand for high resilience (Anthony *et al*., 2011). The recent decline in coral cover on Australia's Great Barrier Reef (GBR) is an example of how increased frequency and severity of pulse-type stressors (a series of severe cyclones, repeated CoTS outbreaks and two extensive coral bleaching events) can overwhelm an ecosystem's resilience (De'ath *et al*., 2012; Table1).

Under business-as-usual carbon emissions, coral bleaching events are predicted to increase in frequency and severity (van Hooidonk *et al*., 2013), and the intensity of tropical storms is likely to be amplified by warming seas in some ocean basins (Knutson *et al*., 2010; Mendelsohn *et al*., 2012). A warming climate thus promotes an increase in globally and regionally driven pulse-type stressors.

#### Press-type stressors

Press-type stressors, such as sustained pollution, sedimentation, overfishing and ocean acidification are key threats to reef resilience (Nyström *et al*., 2008). They influence species sensitivity, rate of coral reef recovery, growth and maintenance, and the interactions between desirable and undesirable system components (e.g. corals vs. fleshy macroalgae). The continuum from low to high press-type stress regimes represents environmental conditions in Fig.1, ranging from unfavourable (e.g. sustained reductions in goods and services) to favourable (resilience processes intact and scope for goods and services). Specifically, on the stability landscapes in Fig.1, press-type stressors act on the system in a direction perpendicular to pulse-type stressors.

Classic examples of press-type stressors with clear management levers reefs are overfishing of herbivorous fish leading to loss of control of macroalgae (Hughes, 1994), enhancement of macroalgal growth via nutrient enrichment (McCook *et al*., 2001) and changing sedimentation and turbidity regimes (Erftemeijer *et al*., 2012). Stressors that have mixed pulse-press characteristics are likely to both reduce resilience and intensify system perturbations (Table1). For example, coral bleaching events triggered by ocean warming reduce coral abundance, growth and reproduction (Baird & Marshall, 2002; McClanahan *et al*., 2012) and increase susceptibility to diseases (Harvell *et al*., 2002). Similarly, sedimentation in coastal waters can occur as dredging events, major run-off events from rivers (e.g. as soil erosion), but also potentially through increased background turbidity regimes (Schaffelke *et al*., 2012). Once large amounts of sediment are deposited in shallow coastal waters, a shift to a pers-istent high-turbidity regime is likely as sediment banks are resuspended by waves and (tidal) currents (Larcombe *et al*., 1995).

### A decision-support framework for ARBM

A key step in operationalizing resilience for management is to identify the ‘levers’ that link to the resilience and vulnerability of the ecosystem and the dependent social systems (Fig.2). The framework presented here builds on adaptive management (Holling, 1978; Schreiber *et al*., 2004; Argent, 2009; Rist *et al*., 2013) in which environmental, ecological and social information is evaluated against management goals and objectives (Fig.2A) and is used as a basis for management decisions (Fig.2B). The framework consists of three broad elements: (i) a management system (Fig.2A–G), (ii) environmental and anthropogenic drivers/activities leading to stress on the ecosystem, which can be influenced to varying degrees by management levers (Fig.2H), and (iii) the linked ecological and social systems (Fig.2I–K). Environmental, ecological and social conditions and impacts all feed back to the management system. As ecological and social systems change in response to stress, the management system records the changes via ecological monitoring, indicators or models (Fig.2C), and via social indicators (Fig.2J). Four avenues for action are possible: (i) managing drivers or activities leading to stress (Fig.2D); (ii) managing stressors directly (E); (iii) supporting ecosystem resilience (F); and (iv) supporting social resilience (G). The degree to which effort and resources should be allocated among these four avenues depends on the environmental, ecological and socio-economic benefits derived from those actions.

#### Setting objectives for successful ARBM

Effective management and decision-making require clear goals and fundamental objectives (Gregory *et al*., 2012), and establishing objectives is the first step of the ARBM framework (Possingham *et al*., 2001) (Fig.2A). The origin of objectives is illustrated by the link between objectives and the social drivers, which are themselves linked to the social- economic benefits derived from the ecological system (Fig.2J–K). To set meaningful objectives, managers need to define what system states are desirable and should be aspired to, and what system states are undesirable and should be avoided (recognizing that conflicts may exist as to what is desirable to whom) and what management intervention is most needed. Objectives and data on system state are hence strongly linked and directly inform decision-making (Fig.2A–C). For coral reefs, high abundance and biodiversity of corals and fish are characteristic desirable states associated with rich goods and services, whereas shifts to macroalgal dominance and a depauperate fish community represent undesirable states (McClanahan *et al*., 2002; GBRMPA, 2009; Hughes *et al*., 2010).

#### Data supporting resilience models and ARBM decision-making

Monitoring of environmental variables and the state and behaviour of the system, and analyses of data and model projections against conservation objectives, all form part of the decision-making process (Nichols & Williams, 2006). Here, the decision framework and linked ecosystem models need to account for the dynamics of the ecosystem and model uncertainty (Carpenter *et al*., 2005; Mumby *et al*., 2014), and to evaluate how the system is likely to be affected by future conditions (Anthony *et al*., 2011). Static measures of desirable states on coral reefs, such as high coral cover and fish abundance and diversity, can be poor indicators of resilience (Mumby *et al*., 2014). High coral cover can be the legacy of past favourable conditions and can fail to alert the decision-maker to declines in resilience, for example reduced recruitment potential or reduced herbivory (Bellwood *et al*., 2004). Such reefs can be prone to a phase shift that might prove difficult to reverse (Nyström *et al*., 2012).

Some state variables can provide information about a range of ecosystem values that underpin resilience. Such variables, termed resilience indicators, are used to substitute simple resilience models (McClanahan *et al*., 2012). Examples of resilience indicators on coral reefs include structural complexity (which supports a rich fauna of fish and invertebrates, Jones *et al*., 2004), coral disease prevalence (McClanahan *et al*., 2012), substrate quality for coral recruitment, including abundance of crustose coralline algae that facilitate coral settlement (Harrington *et al*., 2004), the distribution of important functional groups, such as herbivores (Bellwood *et al*., 2004) and their demographic structure (Nyström *et al*., 2008). Other indicators with close links to resilience processes are competitive strengths between corals and macroalgae (Barott *et al*., 2012) and the abundance and diversity of juvenile corals (Mumby & Steneck, 2008). Also, microbial pathogens are showing increasing potential as early warning systems for stress to coral reef communities (McDole *et al*., 2012).

#### Structured decision-making in ARBM

The decision-making process governs how actions are best identified and implemented to meet objectives based on existing environmental, ecological and social conditions. We integrate the resilience concept with a simple, well-tested system of structured decision-making that has been widely adopted in environmental and conservation planning (Possingham *et al*., 2001; Gregory *et al*., 2012). The decision-making system includes a series of elements condensed into two key groups: (i) data or models of system states and responses to stressors of concern, and (ii) management options or alternatives, and their social, economic and realistic feasibility and consequences. While our framework is applicable in a wide range of settings, managers need to incorporate their specific geographic and socioeconomic conditions, spatial and temporal scales and the system's present status and trajectory. Importantly, managers will need to assess the short and long-term conservation benefits of each option identified through application of the ARBM framework against the financial costs, social impacts and political implications.

We integrate decision-making processes into the ARBM framework by requiring that management actions always attempt to satisfy the fundamental objectives, for example to minimize vulnerability. In the following section, we apply the ARBM framework to coral reef examples in different environmental and socio-economic settings.

### Applying ARBM under local and regional pressures

**Fig 3:**
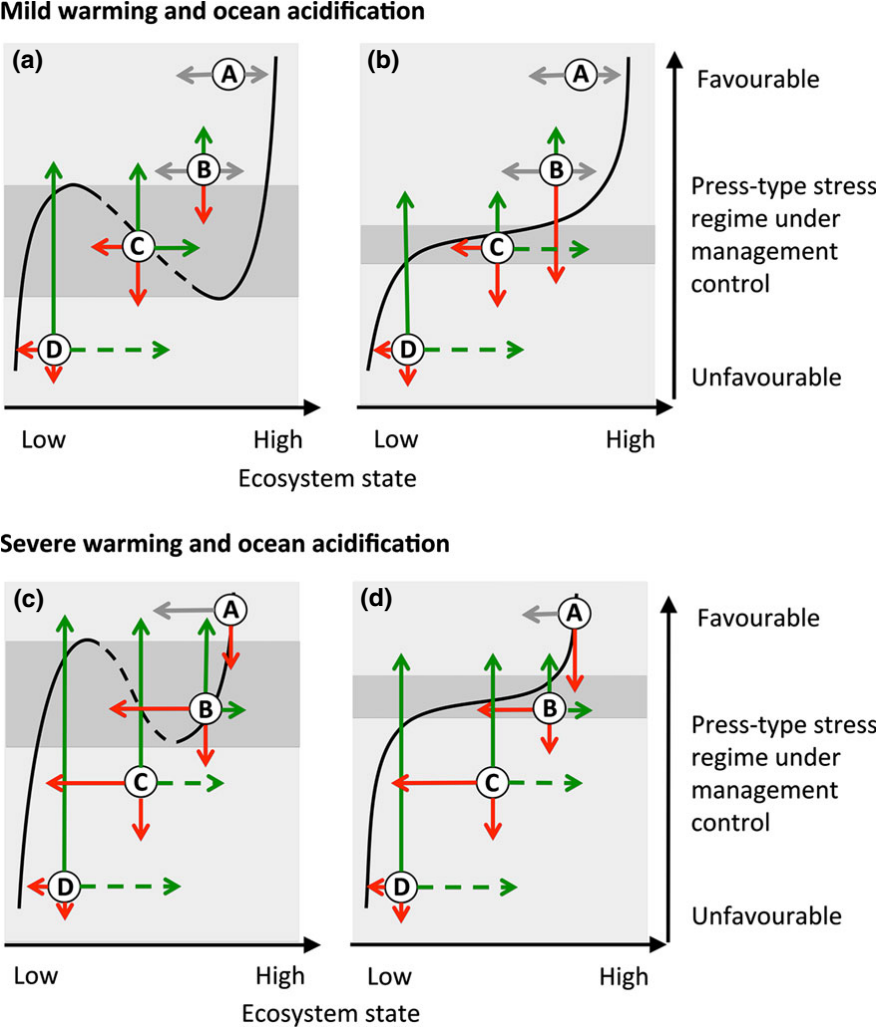
Two-dimensional conceptual representation of system behaviour for the four environmental scenarios and resilience categories (a–d) in Fig.1, and management actions needed to improve system condition and resilience under mild and severe climate change and ocean acidification. Solid lines represent stable equilibria (basins of attraction) and the dashed line the unstable equilibrium (threshold). Grey arrows indicate perturbations or environmental changes that do not represent immediate risks. Red arrows are perturbations that can potentially lead to an unwanted phase shift or reinforce an already low (or degraded) ecosystem state, and green arrows are resilience-based management actions (see also text). The lengths of arrows indicate the severity of disturbances (press or pulse), propensity for recovery or efficacy of efforts needed to move the system to the safe side of a threshold. The grey areas represent the conditions where a pulse-type disturbance may trigger a phase shift. The figure is modified from Figs1 and 2 in Scheffer *et al*. (2001). Thresholds for regime shifts under ocean warming and acidification are guided by model simulations using GBR corals (Anthony *et al*., 2011).

States and environmental settings for coral reefs span the full range depicted in Fig.1. How the ARBM framework is applied to support management decisions under different regional and global environmental scenarios depends in part on the socio-economic setting. To illustrate this, we convert Fig.1 to a two-dimensional representation of coral reef stability landscapes (Fig.3). Specifically, we use the location of thresholds and shape of system equilibria as guidance only. Again, we acknowledge uncertainty and therefore only work with general rules rather than assuming detailed understanding of ecosystem dynamics, variation around equilibria, and the resilience within gravitational basins. Here we first examine how ARBM can be applied to local and regional-scale scenarios assuming mild ocean warming and acidification (Fig.3a and b) and subsequently address how progressed ocean warming and acidification change the actions required to maintain system resilience (Fig.3c and d).

Reefs in the wider Caribbean straddle scenarios A to D in Fig.3a. In general they are characterized by low species diversity and abundance of key groups such as branching hard corals (Burman *et al*., 2012), placing them at the lower end of the resilience spectrum, potentially due to low functional redundancy compared to Indo-Pacific reefs (Roff & Mumby, 2012). The propensity of Caribbean reefs to form alternate stable states has implications for ARBM. In particular, slow erosion of resilience via press-type stress (typically overfishing of herbivorous fish and nutrient enrichment, Mumby *et al*., 2006, 2007) make these systems susceptible to a phase shift triggered by a single pulse disturbance. This is illustrated by a shift from scenario B to C in Fig.3a. Here, critical management actions can be two-pronged. First, efforts to reduce herbivore over-fishing and nutrient run-off (upward green arrow) can shift the system out of the bistable state regime (C in shaded area in Fig.3a) and into the single equilibrial state regime (B). Second, enhanced connectivity through networks of protected areas (Almany *et al*., 2009), local coral restoration (Rinkevich, 2005) and potentially algal removal can potentially push the system (green rightward arrow in scenario C) across the unstable equilibrium (dashed line) from algal to coral domains (Fig.3a). Restoration efforts and other direct control efforts are likely to be effective only at small spatial scales where a particular reef represents substantial goods and services, and in socio-economic settings characterized by a strong sense of stewardship and political responsibility, for example reefs in Florida (Moberg & Ronnback, 2003). However, once the system is degraded to scenario D, i.e. into the single equilibrial basin dominated by macroalgae, no level of restoration or enhanced connectivity can restore coral resilience (green dashed arrow). The most effective ARBM options for scenario D are management actions that reduce press-type disturbances (upward green arrow in Fig.3a, actions D and E in Fig.2). If these measures fail to improve reef condition, adaptation programs that enhance the resilience of reef-dependent communities and explore alternative livelihoods for reef-dependent industries may become the only viable ARBM strategies (action G in Fig.2).

Reef in the Indo-Pacific are generally assumed to display single equilibrial states (Roff & Mumby, 2012). Managing for resilience in these systems needs to be particularly concerned with the environmental set of conditions that represent a threshold for a regime shifts between coral and algal dominance (Fig.3b). The shape and location of state equilibria, and hence the width and steepness of the threshold, vary strongly as a function of the nature and strengths of feedbacks in the system (Mumby & Steneck, 2008; Nyström *et al*., 2008). In general, the prioritization of resilience-based management options for reefs that display single equilibrial states should not differ from those that show alternate stable states if the key ARBM objective is to keep the system in the coral-dominated single-state regime using the precautionary principle (scenario A in Fig.3a and b).

Importantly, reef systems do not occupy specific locations in the stability landscapes, but are likely to have representatives across the landscapes. For example, the condition of Australia's Great Barrier Reef (GBR) ranges between scenario A and D, depending on geography. Water quality (turbidity, sedimentation, nutrients and other pollutants) in inshore regions of the central and southern GBR has declined in pace with intensified agricultural activities in river basins (catchments) draining into GBR waters (Brodie *et al*., 2012). In the context of Fig.3, water quality degradation contributes to the lowering of the suitability of environmental conditions (Fabricius, 2011), i.e. moving the system into a regime with reduced resilience and hence increased likelihood of a shift to an undesirable state (e.g. red arrows from B and C in Fig.3b). Although herbivorous fishes are not targeted commercially on the GBR, declining water quality inshore suppresses herbivore abundance (Cheal *et al*., 2013), potentially lowering the threshold for a regime shift. Also, experimental and correlative evidence suggests that outbreaks of crown-of-thorns starfish (CoTS) are linked to inputs of nutrients into the northern/central GBR, promoting the survival and recruitment of CoTS larvae and increased predation of corals GBR-wide (Brodie *et al*., 2005; Fabricius *et al*., 2010). This is an example of a press-type stressor leading to consequential pulse-type disturbances. Other hypotheses include reduced top-down control of CoTS in fished areas of the GBR (Pratchett *et al*., 2014). GBR reef managers have a variety of ARBM options and management levers available. First, primary long-term management levers are actions on drivers or activities (e.g. land-use and coastal development practices) that alleviate press-type stressors (D in Fig.2). Second, direct control of CoTS can, if effective, reduce the severity of the starfish outbreak (E and F in Fig.2). Thirdly, large-scale spatial planning, including a network of protected areas (Fernandes *et al*., 2005), can help maintain key ecosystem goods and services on priority reefs (points F and I in Fig.2). These actions all contribute to reducing press-type stressors (Table1, green solid arrows in Fig.3b). In addition, direct CoTS control on selected reefs by starfish culling (Kenchington & Kelleher, 1992; Pratchett *et al*., 2014) can, if effective, actively push the system to a higher (coral) state, which in combination with improved water quality can potentially bring the system to, or keep it, on the safe side of the threshold (dashed arrow on C in Fig.3b).

The GBR and Florida represent socio-economic settings where the links between ecosystem conditions and management decisions and actions are strong, where a sense of stewardship prevails, and where resources are available for monitoring and management actions (McCook *et al*., 2010). In contrast, reefs in the Coral Triangle (CT) support different social systems, and exist in contrasting political and cultural environments that influence the setting of management goals and objectives. Coral reefs in the CT are some of the richest and most diverse in the world, but many are severely overfished and subject to pollution from urbanization (Burke *et al*., 2012). Similar to the GBR and the Caribbean, reefs in the CT cover the full span of scenarios in Fig.3a and b, but different local pressures apply and different ARBM solutions are relevant. In areas with low human populations where traditional marine tenure is strong, reef resilience across broad spatial scales are high because of a relatively low level of local threats and a strong sense of stewardship and ownership of marine resources. Conversely, in heavily populated areas, herbivore overfishing, destructive fishing practices and pollution are important causes of resilience loss on coral reefs, and are priority management levers (Table2E). In the context of the ARBM framework, two key impediments to ARBM management actions in heavily populated areas are: (i) intense pressures on the ecosystem from a growing coastal population (Fig.2H), and (ii) a relatively weak sense of stewardship and political responsibility (Fig.2K). Several approaches to ARBM are needed in the CT. Firstly, education and capacity-building of local communities and regional government bodies are critical (Fig.2K, Table2D and G), especially demonstrating how improved reef ecosystem condition can benefit the social-economic system. In addition, economic development and social-political transformations that reduce the external drivers on these factors is essential (Cinner *et al*., 2009).

**Table 2:** Priority levers for adaptive resilience-based management (ARBM) across three geographical and socio-economic examples. Letters D to G refer to intervention points in Fig.2

	Management levers			
Example	D: Influence drivers and/or activities	E: Reduce stressors	F: Support ecosystem resilience	G: Support social-economic resilience
Great Barrier Reef	Influence national emissions policies through education and awareness-raising around climate change and linkages between land use and run-off	Improve land-use management to reduce pollution in receiving waters; maintained fisheries management	Networks of no-take areas (spatial planning for connectivity and population viability of key species); control CoTS at local scales	Work with fishers and tourism operators to help build resilience in their industries
Coral Triangle	Education of local communities andregional government bodies	Reduce fishing of herbivores; stop destructive fishing practices; reduce pollution	Networks of no-take areas (spatial planning for connectivity and population viability)	Capacity-building of local communities and regional government bodies, support alternative livelihoods
Florida Reef System	Education and awareness-raising around climate change and linkages between land use and land run-off	Reduce nutrient and sediment loads; reduce fishing pressure; manage pressures from recreational use	Coral and reef habitat restoration in combination with networks of no-take areas	Work with local communities and the tourism industry to develop adaptation strategies including livelihood transitioning

### ARBM challenges under global environmental change

Ocean warming and ocean acidification are among the most significant long-term threats to coral reefs (Hoegh-Guldberg *et al*., 2007). While global threat reduction is outside the control of managers, local and regional actions can enhance resilience and adaptive capacity locally. The challenge for ARBM, however, is that ocean warming and acidification influence the stability landscape of coral reef ecosystems (Fig.3c and d) by directly impacting on processes that underpin resilience. These include reduced coral growth rates (Reynaud *et al*., 2003; De'ath *et al*., 2009), enhanced competitive strength of algae over corals (Diaz-Pulido *et al.,* 2011), disease risk (Ritchie, 2006), reduced net reef accretion (Silverman *et al*., 2009) and susceptibility to breakage by storms (Madin *et al*., 2008). Further, nutrient enrichment reduces resistance to thermal stress in corals, which exacerbates bleaching risk (Wooldridge & Done, 2009; Cunning & Baker, 2012). The result is a lowered threshold for local-scale press-type stressors such as pollution and reduced herbivory (Anthony *et al*., 2011). This is shown in Fig.3c and d as an upward shift in the environmental threshold. As a consequence, manageable press-type stress conditions that are relatively favourable today may be unfavourable under future ocean warming and acidification. This consequence is illustrated by scenario B in Fig.3. Under mild warming and acidification, reef systems in scenario B are in the coral-dominated regime for both models (Fig.3a and b). Without changes in local stressors or management regimes, these reefs will be captured by the shifting environmental threshold as warming and acidification progress (Fig.3c and d). Similarly, reefs in the bistable regime (Fig.3a) or near the environmental threshold (Fig.3b) under mild warming and acidification, are likely to be shifted into the algal-dominated regime under severe warming and acidification. The implications are that ocean warming and acidification will make it increasingly harder for management actions to maintain reefs in a coral-dominated state (illustrated by upward green arrows in Fig.3c and d). Further, if ocean warming leads to stronger storms (e.g. Knutson *et al*., 2010) and/or more frequent and severe coral bleaching events (e.g. van Hooidonk & Huber, 2009) the future management challenge will also need to overcome the stronger episodic reductions in reefs state (red leftward arrows in Fig.3c and d).

One avenue for dealing with the growing challenge of globally driven stressors in an ARBM context is through a spatial understanding of both pulse- and press-type stress exposures, and consequent spatial resilience and options for management planning (McLeod *et al*., 2012) (Table1). Here, improved fisheries management and the design of marine protected area networks (supporting ecosystem resilience, Fig.1f, Table2F), building on the principles of habitat representation, connectivity and risk spreading (McLeod *et al*., 2009; Grantham *et al*., 2013) can improve the sustainability of coral reefs under local-scale human pressures as well as under climate change. Importantly, however, because the zone of influence for local-scale stressors as well as for their management are a fraction of the global zone of influence of climate change and ocean acidification, managers are likely to be forced to increasingly consider prioritization of reef areas with high intrinsic resilience and/or less disturbance-prone reef areas with high connectivity (Game *et al*., 2008).

Lastly, while global-scale stressors per se can only be addressed at scale through global carbon emissions policies, managers can play an important role in influencing the development of national and global emissions policies by minimizing emissions of management operations and encouraging others to do so through education and by raising awareness (Table2D).

## Discussion

The operational adaptive resilience-based management (ARBM) framework presented here provides a structured approach for incorporating resilience concepts into conservation and natural resource management of coral reefs. Traditionally, biodiversity conservation has been characterized by efforts to reduce a system's exposure to pressures (e.g. Brooks *et al*., 2006). While this is still valid, the ARBM approach provides a lens that explores a broader set of strategic options to sustain resilience in a changing environment and across socio-economic settings.

The ARBM framework, building on adaptive management (Argent, 2009) integrated with resilience principles (Folke *et al*., 2010), bridges the gap between resilience theory and conservation practice by integrating the adaptive management cycle with resilience models (Figs1 and 2). Although widely applicable, the ARBM framework is not designed to provide a recipe for specific management actions. Instead, it is a structure that guides adaptation of management goals and helps identify management strategies that can better accommodate external system drivers and inte-rnal system dynamics under global environmental change.

Climate change and other accumulating global pressures have caused a re-evaluation of the conceptual model that underpins management decisions on coral reefs. In particular, the pervasive and largely inexorable effects of climate change and ocean acidification challenge the expectation that ameliorating local threats will result in preservation of the system in a desirable state. As indicated by the green arrows in Fig.3, climate change and ocean acidification (lower panels) will effectively raise the bar for management efforts as resilience becomes eroded (increased press-type stress) and thresholds for regime shifts are shifted. Importantly, however, if resilience becomes eroded by global pressures, most regional and local-scale management actions can only counteract pressures in a fraction of the zone of influence of ocean warming and acidification. Therefore, there are limits to the extent ARBM can maintain reef resilience under climate change and ocean acidification despite navigating a strategic path on the stability landscape in Fig.3. Also, with limited resources for investment into coral reef management, spatial prioritization (Game *et al*., 2008) and trade-offs of ecosystem goods and services as desirable states are likely to become increasingly relevant under environmental change.

## Conclusions

There is now a robust base of scientific knowledge about the determinants of system resilience of coral reefs (Nyström *et al*., 2008; Anthony *et al*., 2011; McClanahan *et al*., 2012). We apply two alternative stability landscapes for coral reefs to capture generic models across Caribbean and Indo-Pacific reefs, and to provide underpinnings for adaptive resilience-based management across environmental and socio-economic settings. We demonstrate that management of press-type stressors with regional or local-scale levers are the most effective way to enhance resilience, and that driving the system to the safe side of thresholds for regime shifts (whether using the bistable or single equilibrial state model) should be the key objective for ARBM. Where possible, direct action on pulse-type disturbances in addition to remedial action on press-type stressors can be an optimal approach to restoring resilience. Water quality management and direct CoTS control on the GBR, and herbivore fisheries management and reduced nutrient pollution on Caribbean and Indo-Pacific reefs are key examples. Restoration is effective only under environmental conditions within a bistable regime or within a coral-dominated single equilibrial state regime.

In summary, the ARBM framework provides reef conservationists and resource managers with a tool to integrate resilience into decision-making and help prioritize system components for management focus, i.e. management levers. It also enables managers to identify knowledge gaps that are limiting their ability to implement the most effective strategies for reducing system vulnerability. An important application of this framework is the identification of options for increasing overall system resilience by supporting the resilience of industries or communities that depend on ecosystem goods and services – i.e. by facilitating the inclusion of social resilience management into the arsenal of strategies available to coral reef managers. Through application, testing and further development, we believe that this framework will support smarter management actions that in turn will support the resilience of social-ecological systems in a rapidly changing world.
